# Supplementary material for: End Sequence Analysis Toolkit (ESAT) expands the extractable information from single-cell RNA-seq data
Source: Genome Res. 2016 Oct;26(10):1397–410. doi: 10.1101/gr.207902.116 (PMC5052061; doi:10.1101/gr.207902.116)
Supplement: Supplemental Material [file supp_gr.207902.116_Supp_Materials.tar.gz › ESAT-esat_v0.1/web/index.html]

The
ESAT-Quantitation module is an analytical toolkit to measure digital gene
expression (DGE) in end RNA-sequencing libraries. This is a complete re-write
of our old software that vastly improves running speed and has a fixed memory
footprint that is a function only of genome size and not library depth or
complexity. An overview of the analysis performed by ESAT is included below.

The toolkit is implemented in Java and requires version 1.6
or higher. ESAT can be run on a single or multiple samples. When running
multiple samples, all samples are processed together and the results reported
as a matrix. To handle multiple samples you will need to prepare a file
describing the samples indicating the alignment file locations and the
experiment condition assigned to each file. (See ***Usage Examples*** below for
a description of the file format.)

**Download: esat.v1.jar**

**Usage**: 

java -jar esat.jar -task <score3p | score5p>

-alignments <sample description file> |
-in <BAM/SAM alignment file>

-annotations <transcription annotation
file> | -geneMapping <gene-to-transcript map
file>

-out <Output file name prefix> ***<Options>***

|  |  |  |  |
| --- | --- | --- | --- |
| **Tasks:** | | |  |
|  | -task <score3p \| score5p> | | Specifies the type of analysis to perform. ESAT supports two types of libraries (3' or 5') and you can process either a single sample or multiple samples together. Multiple samples are specified using the -alignments option, whereas the single sample is specified by the -alignment option for both 3' and 5' libraries. When processing a single sample use score3p or score5p for 3' or 5' libraries respectively (default is score3p). |
|  |  | |  |
|  |  | |  |
| **Arguments:** | | |  |
|  | -annotations <bed file> | | Points to a transcript annotation file in BED format. If using this option, read alignment start counts will be reported for *each* transcript listed in the file. So, if a gene has multiple isoforms listed in this file, counts will be reported as if each transcript corresponded to a separate gene. |
|  |  | | **OR:** |
|  | -geneMapping <gene-to-transcript  mapping file> | | Points to a gene-to-transcript mapping file whose format is described below. If using this option, all transcripts for each gene are collapsed into a single meta-transcript covering all bases contained in *any* transcript for that gene. This ensures that all isoforms for each gene are represented in the output. The output file references genes by the gene symbol listed in the mapping file, rather than the transcript ID. |
|  |  | |  |
|  | -in <bam/sam file> | | For single sample runs, specifies the path to the alignment BAM/SAM file. |
|  |  | | **OR:** |
|  | -alignments <samples list file> | | A sample file describing all the samples to be processed. This file should contain 2 tab-separated columns, specifying one sample per line. Each line should include:  Column 1: Experiment/condition label (eID). All samples with the same eID will be grouped together as if all data came from the same input file.  Column 2: Sample file path (BAM or SAM format).  The file name must provide the full file name path, and each file ***must*** contain a header. In addition, the segments/chromosomes listed in the header ***must*** match the ones listed in the annotation or gene-to-transcript mapping file. |
|  | -out <filename prefix> | | Specifies the prefix of the output files. Output files will be named <filename prefix>.gene.txt and <filename prefix>.window.txt, containing counts of the number of reads with alignments starting within each *gene* and *window*, respectively. |
|  |  | |  |
|  |  | |  |
| **Options:** | | |  |
|  | **Scanning window parameters:** | | |
|  | -wLen <window length> | Length of the sliding scanning window. (Default=400) | |
|  | -wOlap <window overlap> | Overlap between adjacent sliding windows. (Default=0) | |
|  | -wExt <window extension> | Number of bases past the end (for 3� libraries) or beginning (for 5� libraries) of the transcript to continue the sliding windows. (Note: The actual amount of extension is truncated to prevent collision with neighboring transcripts. If the extension is truncated, a warning message is issued giving both how much the extension was truncated and the gene(s) causing the truncation.) (Default=0) | |
|  | -sigTest <p-value> | Significance test threshold for each window. A SCAN statistic p-value is computed for each window, based on the number of reads starting within the full transcript and the number of reads starting within the window. If a significance p-value is provided, only windows with a p-value less than this threshold are saved. The default setting for this parameter is 1.0, so any windows with non-zero counts are considered �significant�. If multiple significant windows abut or overlap, the range from the beginning of the first to the end of the last abutting/overlapping window is scanned with a window (of the length specified by the �wLen parameter), and the most significant window is located. Only this window is reported. (Default=1.0) | |
|  | -all | As described above, if multiple abutting/overlapping windows have significant counts, only a single, most significant, window is reported. If the �all parameter is supplied, ALL significant windows will be reported. If the �sigTest parameter is not provided, this will result in reporting counts for all windows with non-zero counts. (Default: not selected) | |
|  | -unstranded | Ignores the strand of the alignment and reference, and treats all reads as if they align on the + strand. (Default: not selected) | |
|  |  |  | |
|  | **Alignment quality check:** | | |
|  | -quality <minimum quality> | Minimum alignment quality. Reads with alignment quality less than this threshold will not be processed. (Default=0) | |
|  |  |  | |
|  | **Multimapped** **reads:** |  | |
|  | -multimap <multimap option> | Indicates how multimapped reads are handled. This only applies to BAM/SAM files where the multimapped flag (in the optional fields of the alignment fields section) are provided. Multimapped reads are indicated with NH:<i>, where <i> is greater than 1. Options:  �         normal: treat multimapped reads as normal reads, receiving 1 count for each start location. This results in multiple-counting ambiguous� reads. (Default)  �         ignore: do not count multimapped reads. This avoids double-counting ambiguous reads.  �         scale: counts each multimapped read as 1/<i> count, where <i> is the number of locations to which the read is mapped (provided in the NH:<i> field.) NOTE: Using this option ***greatly*** increases the working memory required for the program, since counts must be recorded in a floating-point array (rather than the default short integer storage). | |
|  |  |  |  |

**ESAT Analysis Overview**

ESAT takes two primary inputs � SAM/BAM
alignment file(s) and a transcript reference file � and identifies locations in
the reference transcripts where a significant number of the aligned reads
start. It identifies these locations as follows:

**Identification of significant window locations:**

�        
First, all of the SAM/BAM
alignment files are scanned, and the total number of aligned reads that start
at each genomic location is counted.

�        
Next, the reference genome
file is loaded. If the gene-to-transcript mapping file is provided (using the �geneMapping parameter), all transcripts
for each gene are collapsed into a single composite transcript containing all
bases in all exonic regions of all transcripts for each
gene. If annotations are provided in a .BED file (with the �annotations parameter),
each transcript is treated separately.

�        
The main scanning window
parameters (described above) define the length of the scanning window (-wLen),
the amount of overlap between windows as the window steps across the transcript
(-wOlap),
and the number of bases that the windows extend past the transcript boundary (-wExt).

�        
Before beginning the scanning
process, intronic regions are removed and the
remaining regions are effectively �spliced� together for windowed counting purposes.
The window is then stepped along, with each window overlapping the previous
window by the amount specified with the �wOlap parameter.
The process is illustrated below:

�        
For each transcript, the
total number of reads is counted. Then, for each window, a family wise error corrected p-value is
calculated based on a SCAN statistic, which specifies how �significant� the
counts in that window are with respect to the total number of reads within that
transcript, under the null hypothesis that reads are uniformly distributed across the transcript. Windows with a p-value less than the threshold specified with the �sigTest parameter are discarded, as
well as any partial windows. (Note: If no significance threshold is specified, the
default threshold of 1.0 is used, and all windows with non-zero counts are
considered significant.)

�        
Finally, if a set of adjacent
(or overlapping) windows all have a significant number of counts, that region
is re-scanned from beginning to end in one-base steps to locate the window
location with the largest number of reads. All of the overlapping significant
windows are then replaced with this single �best� window. (Note: This
re-scanning process can be turned off by using the �all parameter.
If this parameter is selected, counts for ***all*** significant windows will be
reported.)

�        
The locations of all
significant windows are saved for second pass, which counts the reads in each
of these significant windows for each input experiment/condition separately.

**Collection of counts for individual experiments:**

�        
In the second pass, each of
the input alignment files are scanned again, and read counts are collected for
each experiment or condition specified in the alignment file list file
(described below). Read counts are recorded for each experiment/condition and
the read counts are reported in the gene-level and window-level output files
(described below). The gene-level file provides the counts of the total number
of reads aligned to the exonic regions (plus any
extension) of each transcript, and the window-level file provides counts of
reads for each experiment/condition within the significant windows determined
during the first-pass analysis.

**Output
file formats:**

**The gene-level output file** *(<output file prefix>.gene.txt)*
contains a tab-delimited matrix with one row per gene/transcript provided. The
first three columns contain the gene symbol or transcript ID, the chromosome,
and the strand. This is followed by one column for each experiment/condition
indicating the number of reads aligned to locations within the exons of the
gene/transcript for that experiment/condition. The column headers for the first
three columns are: �Symbol�, �chr�, and �strand�. The
column headers for the remaining columns are the names of the experiments (or
conditions) provided in the input file (with the �alignments parameter). If a single input file is provided
with the �in parameter, the data column is named �Exp1�.

**The window-level output file** *(<output file prefix>.window.txt)*
also contains a matrix with one row per window. The first 5 columns contain the
gene symbol (or transcript ID), chromosome, genomic start coordinate, genomic
end coordinate, and strand. This is followed by one column for each
experiment/condition indicating the number of reads aligned to locations within
the exonic regions contained in that window for that
experiment/condition.

For
both files, counts include any extension past the end of the gene/transcript
specified by the window extension (-wExt)
parameter. Partial windows (i.e., windows with length less than the value
specified with the �wLen parameter) are discarded,
with one exception: if the total length of the transcript, plus any extension,
is less than one full window length, read start counts are reported for all
available bases for that transcript.

**Input Files:**

**Alignment
files:**

ESAT takes alignment BAM/SAM files as input. Any alignment
software, such as Tophat, can be used for this
purpose. BAM or SAM files don�t need to be sorted and indexed in order to be
processed, but processing speed might be improved if this pre-processing has
been performed. (See samtools or Picard for tools to
sort and index SAM/BAM files.) The alignment files MUST have headers. BAM or
SAM files don�t need to be sorted and indexed in order to be processed, but
processing speed may be improved if this pre-processing has been performed.

**Annotations file:**

This is the genome
annotation file specified with the -annotations parameter, containing annotations for all
transcripts being evaluated. This file must be in standard BED file format (see
BED format for
details). Note that the genomic coordinates specified in this file must match
those used for the alignment files.

**Gene
Mapping** **file****:**

This
is the gene-to-transcript mapping file provided with the �geneMapping parameter, containing annotations
for all transcripts being evaluated, along with the gene symbol associated with
each transcript. A table containing all required information can be downloaded
from the UCSC Genome Browser website (UCSC Table
Browser) by selecting the species and genome assembly if interest, and the
following additional fields:

�        
***group:*** *Genes and Gene Predictions*

�        
***track:*** *RefSeq* *Genes*

�        
***table:*** *knownGene*

�        
***output format:*** *all
fields from selected table*

Then, provide an output file
name and select �get output�. If the table is downloaded in gzip
format, it must be un-zipped to a tab-delimited text file for input to ESAT. At
a minimum, the gene mapping file must have the following (labeled) columns:

�        
***name:*** *transcript ID*

�        
***chrom******:*** *reference
sequence chromosome (or scaffold)*

�        
***txStart******:*** *transcription start position*

�        
***txEnd******:*** *transcription
end position*

�        
***name2:*** *Gene symbol*

�        
***strand:*** *+ or - strand*

�        
***exonStarts******:*** *exon start positions*

�        
***exonEnds******:*** *exon end position*

If
any of these mandatory columns are missing, the ESAT will terminate with an
error message. Feature coordinates are assumed to be 1-based, �right open� such
that *exonStart*
coordinates point to the base position of the start of an exon and the *exonEnd*
coordinates point to one base after the end of the exon. (Using this coordinate
system, *exonEnd-exonStart**=exonLength*.) *ExonsStarts* and *exonEnds* are
given as a matching set of comma-separated lists of values indicating the start
and end position of each exon relative to *txStart*, similar to the *blockStarts*
feature in BED files.

When
the �geneMapping parameter is selected, all
exons for all transcripts assigned to a given gene symbol are collapsed down to
form a composite meta-transcript whose �exons� cover all exons of all transcripts
for that gene. An example is shown below:

**Alignment file list
file:**

The
file provided with the �alignments parameter is a tab-delimited list of
experiment label-alignment file pairs. Comment lines can be included by staring
the line with the �#� character. An example file is shown below:

# Experiment 1 files:

Exp1� /home/usr1/sample1a.bam

Exp1� /home/usr1/sample1b.bam

# Experiment 2 files:

Exp2� /home/usr1/sample2a.bam

Exp2� /home/usr1/sample2b.bam

Note that multiple alignment files can be included for each
experimental condition. With this example file, two columns of counts would be
provided in the gene-level and window-level output files, with column headers �Exp1�
and �Exp2�.

**Usage
examples:**

**Processing a single .bam file using a .bed file to provide
genomic coordinates of regions of interest:**

java -jar esat.jar -in sample1.bam -annotations genes.bed -out test1

This will use all default parameters (wLen=400, wExt=0, wOlap=0, 3� scoring, stranded, no
quality filtering, treating multmapped reads as
normal reads, and reporting only the most significant, non-zero window within
each set of adjacent non-zero-count windows), and produce two output files: *test1.gene.txt* and *test1.window.txt*.

**Processing
a set of .bam files using a gene-to-transcript mapping file to provide genome
coordinates of transcripts:**

java �Xmx12g �jar esat.jar �alignments
sampleList.txt �geneMapping geneMap.txt �wLen 200 �wExt 1000 �wOlap 50 �sigTest 0.01 �multimap ignore �out test2

The �Xmx12g switch is provided to allocate 12Gb of memory for the Java virtual machine. Alignment files
are listed in the sampleList.txt file (described below), and
the gene/transcript coordinates are given in the geneMap.txt file. If multiple transcripts for a gene are
listed in the geneMap.txt file, all transcripts are collapsed into a single
composite transcript as described in the �Gene Mapping file� section above. The
scanning window width is set to 200 bases with an overlap of 50 bases, and
scanning extends 1000 bases past the 3� end of the transcript. A significance
test is performed for each window, windows with a p-value greater than 0.01 are
discarded, and only the most significant window of length 200 within a range of
overlapping �significant� windows is reported. Any reads that are marked as multimapped are skipped. Two output files, *test2.gene.txt* and *test2.window.txt*, are produced.

A
possible (tab-delimited) sampleList.txt file is shown below. Note
that lines beginning with the comment character �#� are ignored.

**Warning/error
messages:**

**Extension warnings:**

If
the �wExt parameter is set a value greater than 0, it is
possible that when attempting to extend past the end of a transcript, the extension
would collide with a neighboring transcript. If this occurs, the region is
extended only as far as possible without overlapping the neighboring gene, and
reported with a warning such as:

�WARN
� Gene ENSMUSG00000077212.1 (-) extension overlaps ENSMUSG00000042354.6\_5.
400-base extension shortened to 59�

This
indicates that, when attempting to extend the 6th exon of the transcript
ENSMUSG00000042354.6 (the �\_5� indicates the zero-based exon ID) by 400 bases,
it collided with the transcript for ENSMUSG00000077212.1 and had to be
truncated to 59 bases in order to avoid the collision.

**Isoform mismatch warnings:**

When
a transcript-to-gene file is provided with the �geneMapping
parameter, all transcripts for each gene are collapsed into a single composite transcript,
as described in the **Gene Mapping file** section above. The transcripts of
some genes include copies of that gene on other chromosomes, rather than just
additional isoforms (splice variants) of that gene. For simplicity, the first
transcript listed for each gene is assumed to have the �correct� chromosome for
that gene. If transcripts of that gene listed later in the file appear on a
different chromosome, they are rejected with a warning message such as:

�WARN
� New isoform mismatch for Gm5512 (chr10+) with NR\_002891 (chr19+)�

If this causes problems for your analysis, the
gene-to-transcript mapping file can be edited with this in mind. For example,
if there are two known copies of gene *X*
one two different choromsomes, and both copies are of
interest to your analysis, the file could be modified such that the gene symbol
for all transcripts on chromosome A are *X.chrA*, and all transcripts on chromosome B are named *X.chrB*. If it
should turn out that the �true� main isoform of a gene of interest is not the
first transcript listed in the file, simply modify the file to put the main
isoform first.

**Count overflow
warnings:**

The counts of the number of reads starting at each genomic
location are stored in unsigned short integer (16-bit) arrays in order to minimize
the memory footprint of ESAT. If the number of reads with alignments starting
at a particular location exceeds the maximum possible number that can be stored
(65,535), it is flagged with a warning message such as:

�WARN � location 14238898 in chr7 (-) has >65535 counts.�

Each overflow location is only flagged once, and counts for
that location will then be saved as floating-point numbers so that the correct
number of counts at that location is recorded.
